# Borderline personality disorder in adolescents as a predictor of social anxiety

**DOI:** 10.1192/j.eurpsy.2024.971

**Published:** 2024-08-27

**Authors:** L. Baranskaya

**Affiliations:** Psychiatry, Psychotherapy and Narcology, Ural State Medical University, Yekaterinburg, Russian Federation

## Abstract

**Introduction:**

Borderline personality disorder (BPD) is a mental disorder characterized by unstable relationships, a tendency to self–destruction, affective and behavioral dysregulation and BPD are a clinical problem

**Objectives:**

Early detection and timely intervention for BPD is becoming a new public health priority as it helps prevent the adverse personal, social and economic consequences of the disorder. Borderline personality disorder first manifests itself, as a rule, in adolescence, so it is easy to mistake it for manifestations of “difficult age” characteristic of the period of growing up. In this sense, the typical signs of borderline personality disorder are not original: low self-esteem, emotional excitability, impulsive behavior and sudden mood swings, to one degree or another characteristic of all adolescents. An alarming exception is, perhaps, only a tendency to self-harm and, the so-called, desocialization of a teenager, the loss of social skills and connections (for example, friendships). Recently, experts have increasingly mentioned desocialization in connection with the development of Internet technologies and gadgets that replace communication in real life for many teenagers

**Methods:**

An anonymous survey of 57 older teenagers conducted. The degree of borderline personality disorder assessed using IPDE, STAI, and CDI. Statistical processing of the results carried out in Microsoft Excel using measures of the central trend (arithmetic mean, standard deviation) and correlation analysis. The significance of the differences between the groups was determined using the Student’s t-test (p < 0.05)

**Results:**

On average, the level of BPD among the respondents was at a low level of 9.81 (±4.43) points. The severity of personal anxiety was at a high level of 45.02 (±13.25) points, situational anxiety was also at a high level of 41.14 (±14.93). The severity of depression was above average and amounted to 55.84 (±14.33) points

**Conclusions:**

Teenage girls are more prone to anxiety and depression than boys are. High anxiety causes a tendency to depression, and these two factors affect the occurrence of PRL. The average score does not affect the manifestations of anxiety, depression and the occurrence of BPD

**Disclosure of Interest:**

None Declared

